# Influence of intensity of exercise training on physical performance and myocardial morphology of female rats with type I diabetes mellitus

**DOI:** 10.1186/1758-5996-7-S1-A142

**Published:** 2015-11-11

**Authors:** Edson da Silva, Bárbara Lúcia Santos Soares, Larissa Silveira Ferreira, Cynthia Fernandes Ferreira Santos

**Affiliations:** 1 Universidade Federal Dos Vales do Jequitinhonha e Mucuri – UFVJM, Diamantina, Brazil

## Background

Diabetic cardiomyopathy is associated with cardiac muscle remodeling, resulting in myocardial dysfunction, whereas physical exercise is an important strategy for the management of diabetes mellitus (DM).

## Objectives

This study aimed to investigate the influence of high-intensity and low-intensity training on the structural remodeling of the heart in rats with unmanaged experimental Type 1 DM.

## Materials and methods

Ninety-day-old female Wistar rats were divided into three groups: exercised-control (EC; n=5), high intensity exercised-diabetic (HIED=high intensity training, 80% of the maximum speed in the stress test; n=4) and low intensity exercised-diabetic (LIED=low Intensity training, 40% of the maximum speed in the stress test; n=6). The diabetes was induced in the rats by administration of Alloxan monohydrate Sigma (ALX, 50 mg kg-1 BW). Fatigue strength test and the maximal exercise test were performed before DM induction. Fatigue strength test consisted of treadmill running at 20m/min with slope equal to 0° until the animal could not run spontaneously. Time and distance were determined at the end of the race. Maximal exercise test consisted of treadmill running with a load of 5m /min every 3 min until the animal could not run spontaneously, when it was determined the maximum load. Animals ran on a treadmill running 1 hour/day, 5 days/week for 6 weeks with a load (high or low intensity). After this period animals were sacrificed and hearts removed, weighed and prepared for histomorphometric analysis.

## Results

Six weeks after ALX induction, blood glucose in the HIED and LIED groups were greater (p < 0.05) than EC group. HIED group showed increase (p< 0.05) of maximum speed of the effort test. Regarding the fatigue strength test only HIED group showed greater total test time (p< 0.05). Cardiomyocytes density of diabetic groups had higher values compared to the EC (p<0.05). Diabetic animals showed cardiac hypertrophy and this is most significant in the HIED group (p< 0.05). The myocardium of diabetic rats had increased fibrosis (p< 0.05). More importantly, these tissue fibrosis were attenuated by high intensity training.

## Clonclusion

Physical training favored the physical fitness and attenuated heart pathological changes of the animals, which are more significant in the HIED group. Furthermore, physical training at different intensities appears to have differential effects on bone histomorphometric parameters of diabetic animals.

